# The diverse effects of transforming growth factor-β and SMAD signaling pathways during the CTL response

**DOI:** 10.3389/fimmu.2023.1199671

**Published:** 2023-06-23

**Authors:** Karthik Chandiran, Linda S. Cauley

**Affiliations:** ^1^ School of Biology, Indian Institute of Science Education and Research, Thiruvananthapuram, Kerala, India; ^2^ Department of Immunology, UCONN Health, Farmington, CT, United States

**Keywords:** cytotoxic T lymphocytes (CTL), adhesion molecules, transforming growth factor beta, CD8 memory T lymphocytes ^+^, SMAD4, CD8 T cell differentiation

## Abstract

Cytotoxic T lymphocytes (CTLs) play an important role in defense against infections with intracellular pathogens and anti-tumor immunity. Efficient migration is required to locate and destroy infected cells in different regions of the body. CTLs accomplish this task by differentiating into specialized subsets of effector and memory CD8 T cells that traffic to different tissues. Transforming growth factor-beta (TGFβ) belongs to a large family of growth factors that elicit diverse cellular responses via canonical and non-canonical signaling pathways. Canonical SMAD-dependent signaling pathways are required to coordinate changes in homing receptor expression as CTLs traffic between different tissues. In this review, we discuss the various ways that TGFβ and SMAD-dependent signaling pathways shape the cellular immune response and transcriptional programming of newly activated CTLs. As protective immunity requires access to the circulation, emphasis is placed on cellular processes that are required for cell-migration through the vasculature.

## Introduction

**Figure 1:**
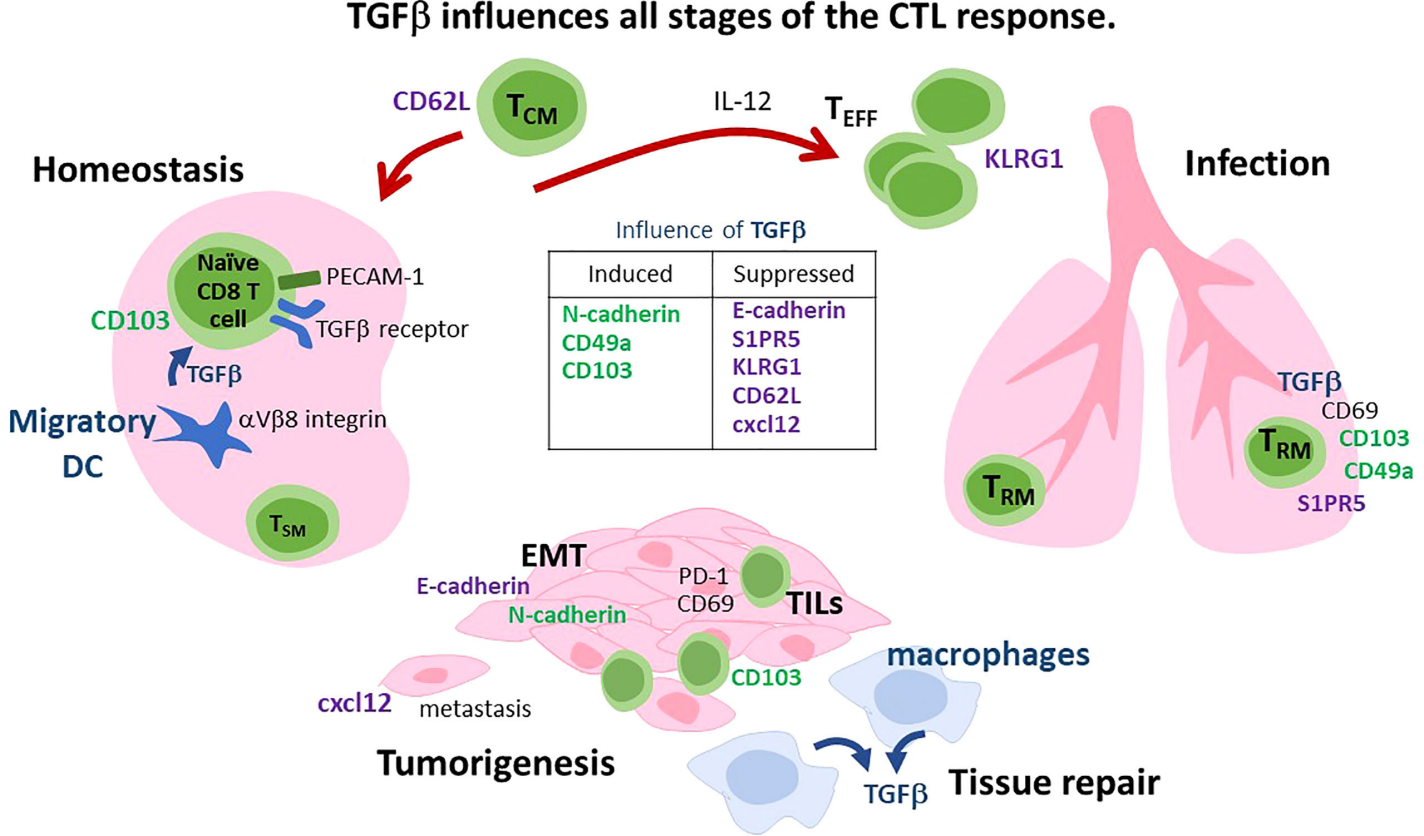
Diverse functions for TGFβ during the CTL response. i) Homeostasis: PECAM-1 is expressed on naïve CD8 T cells and interacts with the TGFβ receptor to inhibit autoimmunity. Some naïve CD8 T cells are preconditioned to become T_RM_ cells by migratory DC that express αVβ8 integrin and activate TGFβ. CD62L is lymphoid homing receptor that can be downregulated by TGFβ. ii) Infection. IL-12 overrides the suppress effects of TGFβ to enhance T_EFF_ and T_CM_ formation. T_RM_ cells express adhesion molecules (CD103 and CD49a) that are induced by TGFβ, while S1PR5 is downregulated. Signaling via CD69 increases TGFβ production in the spleen. iii) Tumorigenesis and tissue repair. TGFβ is an angiogenic factor that promotes tumor growth and tissue repair. During EMT, TGFβ induces a ‘cadherin-switch’ by downregulating E-cadherin and inducing N-cadherin expression on mural cells. Macrophages are an important source of TGFβ during tissue repair. Specialized subsets of CTLs express cadherin-binding proteins (KLRG1 and CD103) and reside at barrier surfaces. CD103^+^ T_RM_ cells mobilize from draining lymph nodes to the tumor when TGFβ signaling is disrupted. CTLs are excluded from tumors when CXC12 is expressed. Cancer cells undergo EMT in the presence of TGFβ. CXCL12 can be downregulated by TGFβ.

Transforming growth factor beta (TGFβ) is the signature cytokine for a large family of growth factors. The three isoforms of TGFβ (TGFβ − 1, 2 & 3) are encoded by separate genes and produce structurally similar cytokines (1). These cytokines use autocrine and paracrine signaling pathways to control a diverse range of biological processes that support immune cell function and tissue repair (2). TGFβ is secreted as an inactive complex with the latency associated peptide (LAP), that binds to the extracellular matrix (ECM) or a receptor (glycoprotein A repetitions predominant, GARP) that is expressed on regulatory T cells (Tregs) and platelets (3). Biologically active TGFβ is released from reservoirs in peripheral and lymphoid tissues when proteases or adhesion molecules induce steric changes in the conformation of LAP (4, 5). The active cytokine is a charged peptide with a short half-life that limits biological activity to the local tissues (1). TGFβ interacts with a bivalent transmembrane receptor with serine/threonine kinase activity, that elicits cellular response via a network of interconnected signaling pathways (6). Canonical signals are mediated by a cascade of structurally-related signaling intermediates known as SMAD proteins (7). SMAD4 is an adaptor for multiple pathways within the SMAD cascade (8). Multiple SMAD-dependent signaling pathways coordinate changes in homing receptor expression as cytotoxic T lymphocytes (CTLs) respond to infection (9–11). The various ways that TGFβ impacts the CTL response depends on the timing and context of cytokine exposure. In this review, we examine the influence of TGFβ at different ages during the CTLs response (**Figure 1**), starting with naïve CD8 T cells, followed by effector CD8 T cells (T_EFF_) and specialized subsets of memory CD8 T cells that are adapted to circulate in the blood, or enter peripheral tissues.

## TGFβ is involved in early grooming of adolescent T cells

Secondary lymphoid organs (SLO) are surveyed by quiescent populations of naïve CD8 T cells searching for early signs of infection. Although homogeneous surface markers indicate limited functional diversity, transcriptome analysis reveals extensive genetic variability as clonal populations of naïve CD8 T cells respond to cognate antigen. Studies show that environmental stimuli play an important role in transcriptional programming of naïve CD8 T cells during transit around the body. Transient interactions with Major histocompatibility complex class I molecules (MHCI) extend the lifespan of naïve T cells through tonic activation of the T cell receptor (TCR). The circulating cells encounter different assortments of peptides according to the tissue of origin and mechanisms that are used for antigen processing. The impact of TCR stimulation varies according to the affinity of interactions with self-peptides as well as availability of costimulatory signals and exposure to local inflammatory conditions (12, 13). In lymphoid tissues, naïve CD8 T cells are preconditioned to become tissue resident memory CD8 T cells (T_RM_) during interactions with migratory dendritic cells (DCs) that activate TGFβ (14).

Fate-mapping techniques underscore the importance of environmental stimuli during CD8 T cell differentiation. A study of naive CD8 T cells isolated from different tissues showed variable capacities to proliferate and differentiate into specialized subsets of memory CD8 T cells (15). Similarly, naive CD8^+^ T cells that were isolated at different times after birth showed that early microbial exposure has long term effects during the CTL response (15, 16). CTLs that were derived from newborn mice rapidly differentiated into T_EFF_ cells, whereas CTLs from adult animals were more inclined to become memory CD8 T cells under similar conditions (15, 16). The effects of chronic exposure to inflammation also contribute to age-related changes in protective immunity (17). Characteristics of naïve CD8 T cells in elderly people include diminished capacity for T_EFF_ and memory formation (18, 19). Several mechanisms contribute to suboptimal CTL responses during ageing, including immune cell senescence and increased production of TGFβ (20, 21). Responses to TGFβ vary at different stages during the CTL response, as revealed by changes in cell proliferation, survival and memory formation (22–24).

CD8 T cells express a variety of inhibitory receptors (IR) that help maintain diverse repertoires of functional CTLs under homeostatic conditions. Platelet endothelial cell adhesion molecule-1 (PECAM-1 or CD31) is an adhesion molecule with an immunoreceptor tyrosine-based inhibitory (ITIM) motif in the cytoplasmic tail (25) and forms homophilic interactions between endothelial cells to maintain barrier integrity. PECAM-1 is also expressed on cells in the vascular compartment, including naïve CD8 T cells and cooperates with the TGFβ receptor to inhibit autoimmunity during stimulation with self-antigens (24, 26) by enhancing non-canonical (SMAD-independent) signaling (27). PECAM-1 is transcriptionally downregulated during formation of short-lived T_EFF_ cells (SLECs), whereas expression is maintained in memory precursors (28). In the absence of PECAM-1, T_EFF_ cells become resistant to the suppressive effects of TGFβ during production of IFN-γ, and granzyme B, as well as cell-proliferation. Naïve CD8 T cells express other receptors that inhibit responses to self-peptides, including V-domain Ig suppressor of T cell activation (VISTA) (29). A study found that exogenous TGFβ decreased VISTA expression on human CD8 T cells *in vitro *(30). P-selectin glycoprotein-1 (PSGL-1) is another adhesion molecule that is expressed on endothelial cells and interacts with VISTA to inhibit TCR activation and IL-2 production (31, 32). During LCMV infection, increased numbers of CD4 T cells expressed PSGL-1 in the absence of the TGFβ receptor (33), although the study did not reveal whether a similar change occurred in CD8 T cells.

## The influence of TGFβ during angiogenesis and tissue repair

Blood is transported around the body through an elaborate network of interconnected vessels. Extracellular fluid drains from peripheral tissues into blind-ended lymphatic vessels that join the blood supply at the thoracic duct (34). The vessels are lined with a tightly connected layer of endothelial cells that provide a physical barrier between the circulation and surrounding tissues (35). Cutaneous and mucosal surfaces are covered with similar layers of stromal cells that provide a barrier against infection (36). Contacts between adjacent stromal cells are mediated by protein complexes called tight junctions (TJs) and adherens junctions (AJ). AJs are mediated by calcium-dependent adhesion molecules, known as cadherins (35, 37). Migrating T cells use specialized homing receptors to penetrate the stromal layer and enter the vascular system during immune surveillance.

During infection, barrier integrity can be comprised by high concentrations of proinflammatory cytokines that disrupt connections between adjacent stromal cells (38). In the respiratory tract, damaged bronchial epithelial cells are released from the basement membrane and expelled from the lungs by the mucociliary escalator (39). As inflammation resolves, macrophages produce large quantities of TGFβ to facilitate tissue repair (40, 41). TGFβ endows epithelial cells with migratory properties by triggering epithelial-mesenchymal transition (EMT) (42). A ‘cadherin-switch’ occurs as TGFβ downregulates epithelial (E)-cadherin (43–45) and induces neural (N)-cadherin expression (40). During angiogenesis, TGFβ converts endothelial cells to mural cells via a similar process (35, 46, 47). AJs between endothelial cells are mediated by vascular endothelial (VE)-cadherin, whereas N-cadherin is diffusely expressed over the surface of endothelial and mural cells including pericytes (48, 49). As CTLs mobilize to inflamed tissues, PECAM-1^+^ T_EFF_ cells disrupt AJs by dephosphorylating VE-cadherin to transit across the blood vessel wall (50). Pericytes that reside between endothelial cells and the basement membrane around capillaries play a central role in angiogenesis (51).

## Cytokines program CTLs for specialized functions

During priming, naïve CD8 T cells participate in serial interactions with DCs (52) that provide costimulatory signals to augment T_EFF_ functions and memory formation (53). Initial interactions with DCs occur in the interfollicular and cortical regions of the draining lymph node (54). After a few rounds of cell-division, activated CTLs join clusters of DCs that express chemokine receptor XCR1 and CD8 (53, 55). TGFβ that is activated by migratory DCs that express αV integrin conditions naive CD8 T cells to become T_RM_ cells (14). After proliferation, activated CTLs enter the circulation and distribute around the body. The properties of migrating T cells can be modified through interactions with endothelial cells that express costimulatory molecules (CD40, ICOSL, 4-1BB, and OX40L), or ligands of IRs such as PD-L1 and PD-L2 (56). Antigen-presentation on endothelial cells promotes extravasation of CTLs from the blood vessels to adjacent tissues.

Cytokines have important regulatory functions during the CTL response (57). During the acute phase of infection, interleukin IL-12 and other inflammatory molecules initiate a transcriptional program that leads to T_EFF_ formation (58). T cell factor 1 (TCF1) is a transcription factor that programs stem-like memory (T_SM_) cells for multipotency and self-renewal (59) and enables partially exhausted CTLs to maintain some T_EFF_ functions during chronic infection (60). During the T_EFF_ response, IL-12 downregulates TCF1 (61), as Tbet and LFA-1 are induced (62, 63). Most short-lived T_EFF_ cells undergo apoptotic cell death as the infection comes under control, leaving heterogeneous populations of memory CD8 T cells in the circulation and infected tissues. TGFβ has opposing effects on T cell survival at specific stages during the CTL response (24, 64). As inflammation subsides, the IL-12 receptor is down-regulated by TGFβ as T_RM_ cells settle in peripheral tissues (65, 66). T-bet prolongs survival of T_RM_ cells in the skin by upregulating the receptor for IL-15 (67).

T_EFF_ cells produce large quantities of pathogenic cytokines (IFNγ and TNFα) and lytic molecules that contribute to organ damage during severe infections (68–70). Local concentrations of inflammatory molecules must be tightly controlled to restrict immune pathology in the lungs. Killer cell lectin-like receptor G1 (KLRG1) is a membrane-bound adhesion molecule that is expressed some subsets of NK cells, Tregs and CTLs (71). Inflammatory mediators that are released during microbial infection are responsible for massive up-regulation of KLRG1 on CD8^+^ T_EFF_ cells (72). Although large numbers of newly activated CTLs transiently express KLRG1 during antigen-stimulation (73), stable expression is a feature of terminally-differentiated CTLs that do not convert to a memory phenotype (74). Several studies found long lived KLRG1^+^ CTLs in the vasculature and spleens after infections with different pathogens (75–77). After transfer, these CTLs provided robust protection against infection with *Listeria monocytogenes* (LM) through a robust lytic response and IFNγ production (76). Prolonged survival of KLRG1^+^ CTLs after LCMV infection required Treg-derived IL-15 (78).

PSGL-1 and KLRG1 are IRs with ITIM sequences in the cytoplasmic domain (79). KLRG1 interacts with two members of the cadherin family (E- and N-cadherin) that are expressed on different types of stromal cells and differentially regulated by TGFβ (79, 80). While the function of KLRG1 is poorly understood, crosslinking with anti-KLRG1 antibodies impaired TCR-mediated Ca2+ mobilization and cytolysis in CTLs that were engineered to express KLRG1 at high levels (81). The same study found that cross-linking antibodies also decreased IL-2 production from cell lines that over expressed KLRG1.

Infection with LM elicits a robust inflammatory that favors formation of T_EFF_ that express KLRG1. A study found that the frequency of these terminally differentiated CTLs increased when the TGFβ receptor was not expressed (82). Although KLRG1^+^ CTLs can produce large quantities of lytic molecules and proinflammatory cytokines, intranasal infection with LM-OVA does not cause lethal pathology in the lungs, irrespective of whether the TGFβ receptor is present (9). It possible that inhibitory signals that are mediated via KLRG1 protect cadherin-positive endothelial cells from cytolysis and thus minimize damage to capillaries during the T_EFF_ response. A specific mechanism that is responsible for retaining KLRG1^+^ CTLs in the vasculature has been not identified, however these CTLs express chemokine receptor CX_3_CR1 at high levels which may influence tissue localization (9, 83). Potential functions of blood resident CTLs during protective immunity include recruitment of circulating immune cells to inflamed tissues by altering vascular permeability.

## Migrating CTLs enter the vasculature and commute to work

Multiple subsets of memory CD8 T cells use the bloodstream to circulate around the body. Central memory CD8 T cells (T_CM_) express lymphoid homing receptors [CD62L & chemokine receptor 7 (CCR7)] and enter SLO, whereas effector memory CD8 T cells (T_EM_) cells express molecules that aid localization to inflamed tissues. Although T_EM_ cells are excluded from resting peripheral lymph nodes (pLN), some cells enter reactive pLN during the resolution of the immune response and help restore homeostasis by destroying antigen-bearing APCs (84). As inflammation declines TGFβ counteracts the effects of IL-12 (82) to promote development of T_CM_ and non-circulating T_RM_ cells (66, 85, 86). Under resting conditions, constant signaling via the TGFβ receptor shapes the memory compartment (87) and limits homeostatic proliferation by increasing sensitivity to IL-7 and IL-15 (88). The circulating pool of memory cells also includes some stem-like memory CD8 T cells (T_SM_) that express TCF1 and differentiate into mixed populations of T_CM_ and T_EM_ cells after antigen stimulation (59, 89). TCF1 inhibits T_RM_ formation by inhibiting TGFβ-induced CD103 expression (85). During chronic infection, TCF1 induces a transcriptional program that preserves T_EFF_ functions of exhausted CTLs by upregulating FOXO1, ZEB2, Id3, and Eomesodermin (EOMES) (60). TGFβ enforces the stem-like properties of PD-1^+^ T_SM_ cells in lymphoid tissues by inhibiting T_EFF_ formation (90, 91).

T_CM_ cells follow similar migratory patterns to naïve CD8 T cells and enter pLN from wide blood vessels known as high endothelial venules (HEVs) (34). Selectins are vascular adhesion molecules that aid leukocyte migration to peripheral tissues (92). During transit through HEVs, circulating T cells that express (L)-selectin (CD62L) adhere to peripheral lymph node addressin (PNAd) expressed on cuboidal endothelial cells (34). After T cells are tethered to the blood vessel wall, ligands of CCR7 trigger an adhesion cascade that results in trans-endothelial migration (93, 94). PSGL-1 interacts with selectins expressed on activated endothelial cells and initiates a similar adhesion cascade as T_EFF_ cells extravasate to infected tissues (95, 96). T_CM_ cells (but not naive T cells) are also able to access pLN via a CCR7-independent mechanism that involves CXCL12/CXCR4 (97). Although CD62L is associated with immune surveillance in SLO, this molecule also facilitates recruitment of CTLs to inflamed tissues (98). Endothelial cells that line HEVs express a leucine-rich HEV glycoprotein (LRHG) that binds to latent TGFβ (99). Downregulation of CD62L by TGFβ (9) may limit recruitment of circulating T cells to reactive pLN and inflamed tissues during tissue repair.

Chemokine receptors are dynamically regulated during the CTL response and aid localization in different tissues. Several receptors are regulated by TGFβ including CXCR3 which is expressed on T_EFF_ cells during mobilization to inflamed tissues (100). A study showed that TGFβ reduced CXCR3 expression on T_EFF_ cells and prevented tumor infiltration via a SMAD2-dependent pathway (101). The fractalkine receptor (CX3CR1) is also absent from naïve CD8 T cells and induced during the T_EFF_ response. Whereas T_CM_, T_EM_ and T_RM_ cells all lack CX3CR1 (102), expression is maintained on long lived KLRG1^+^ T_EFF_ cells in the vasculature (9). Additional memory CD8 T cells that express CX3CR1 at intermediate levels, exhibit features of both T_CM_ and T_EM_ cells and are primarily responsible for immune surveillance in non-lymphoid tissues (102, 103).

## Remote workers enhance efficiency as CTLs respond to infection

Cutaneous and mucosal surfaces exposed to a diverse array of pathogens that propagate in human cells. Barrier tissues are densely populated with T_RM_ cells that leave the circulation and become lodged in the local tissues where they are poised for a rapid response upon infection (104). T_RM_ cells reside in virtually all tissues in the human body (105) and augment immunity by releasing inflammatory molecules that attract circulating immune cells to the site of infection (106). These cells have limited capacity for self-renewal and proliferation after antigen stimulation and can be divided into subsets based on variations in surface receptor expression. The canonical markers of T_RM_ cells include two members of the integrin family that direct localization in peripheral tissues. α1β1 integrin (CD49a) is induced by stimulation with either IL-12 or TGFβ, and aids localization in tissues that contain collagen (107, 108). Conversely, αEβ7 integrin (CD103) is induced by TGFβ and negatively regulated by IL-12 during the T_EFF_ response (66). Interactions between CD103 and E-cadherin enhance retention of T_RM_ cells in tissues with an epithelial layer and stabilize interactions with target cells during cytolysis (109). Although CD103 is expressed on a majority of T_RM_ cells at barrier surfaces and in glandular tissues, it is not a universal marker of T_RM_ cells in all tissues (110). In the liver, T_RM_ cells develop independently of TGFβ and lack CD103 expression (110). TGFβ upregulates CD103 expression on naïve CD8 T cells, but not T_CM_ cells (111). After reactivation, T_CM_ cells give rise to CD69^+^ T_RM_ cells that do not express CD103 in the presence of TGFβ (111, 112).

Sphingosine-1-phosphate (S1P) is a bioactive lipid that is present in blood and lymph and attracts migrating leukocytes into the circulation. Different S1P receptors (S1PR) are required for CTLs to leave peripheral and lymphoid tissues and are downregulated on T_RM_ cells to prevent entry into the circulation (113, 114). CD69 is a C-type lectin that is transiently induced during antigen stimulation and prevents newly activated CTLs from leaving SLO during clonal expansion, by modulating S1PR1 from the cell surface (115, 116). Lymphoid T_RM_ cells that express CD69 remain in reactive pLN while S1PR1 is down regulated (114). S1PR5 is required for CTLs to leave peripheral tissues and is downregulated by TGFβ during T_RM_ formation (114). An arthritis model showed that signaling via CD69 reduced immune reactivity by stimulating TGFβ production from mouse splenocytes and transformed CD8 T cells (117). Large numbers of T_RM_ cells in peripheral tissues maintain CD69 expression in the absence of antigen stimulation. While it is unclear how this phenotype is enforced, CTLs acquired properties of T_RM_ cells and upregulated CD69 *in vitro* during interactions with activated endothelial cells (118). CD69 is not required for T_RM_ development in some tissues (119).

During tissue remodeling, TGFβ induces a ‘cadherin switch’ by altering E- and N-cadherin expression (40, 42). KLRG1 and CD103 are cadherin-binding proteins that are transcriptionally regulated by TGFβ (9). Whereas KLRG1 is down-regulated by TGFβ (9, 82) CD103 is induced (120) and opposing signaling mechanisms prevent dual expression on the same CTLs (9). When a retroviral vector was used to force KLRG1 expression in newly activated CTLs, reduced numbers of CD69^+^CD103^+^ T_RM_ cells accumulated in the skin of HSV infected mice (121). It is unclear whether the ITIM sequence in the cytoplasmic domain of KLRG1 prevented T_RM_ development, or whether dysregulated homing receptor expression promoted localization in other tissues.

## TGFβ has positive and negative effects on tumor progression

Therapeutic approaches for cancer treatment include strategies that are designed to elicit robust responses from tumor specific CTLs. Although entry into the tumor microenvironment is a critical step in immune control, only small numbers of circulating CTLs home to solid tumors. Efforts to augment antitumor immunity by vaccination have been hindered by limited knowledge of the mechanisms that determine how CTLs distribute to different tissues.

TGFβ is a multifaceted cytokine that has beneficial and detrimental effects during cancer treatment. The protective effects of TGFβ include suppression of cancer cell-proliferation and retention of CD103^+^ T_RM_ cells in the tumor micro-environment. The presence of CD103^+^ T_RM_ cells in tumors is associated with positive prognoses for several types of cancer (122, 123). Murine models show that T_RM_ cells delay tumor progression by releasing cytokines (Interferons and TNFα) that attract other immune cells into the tumor microenvironment.

During cancer treatment, the therapeutic effects of TGFβ are outweighed by its proangiogeneic properties that support tumor growth and capacity to promote metastatic disease through induction of EMT (40, 124). TGFβ also contributes to dysfunctional responses by tumor specific CTLs. Cell mediated immunity is compromised as tumor infiltrating lymphocytes (TILs) respond to continuous antigen stimulation by upregulating IRs and progressively lose the capacity to produce cytokines and destroy tumor cells (125). TGFβ contributes to the functional impairment of TILs by altering the expression levels of several IRs including programmed death protein-1 (PD-1) and cytotoxic T lymphocyte antigen-4 (CTLA-4) (122).

A prostate cancer model was recently used to divide the anti-tumor response into stages. During the induction phase tumor specific CTLs were primed by APCs in the surrounding pLN (126, 127). After antigen-stimulation, stem-like CTLs mobilized to the tumors and acquired T_EFF_ functions after receiving additional costimulatory signals (126). Several studies found that tumor draining lymph nodes contained stem-like CD8+ T cells that converted to CD103^+^ T_RM_ cells in the presence of TGFβ (91, 127, 128). Importantly, disruption of TGFβ signaling enhanced cell mediated immunity by converting lymphoid T_RM_ cells to T_EFF_ cells that localized in the tumor (91). Others induced a robust antiglioma response in mice that were vaccinated with tumor cell-lysate, using a compound that inhibits TGFβ-signaling (129).

Immune checkpoint blockade (ICB) is a strategy that is used to reinvigorate the responses of partially exhausted CTLs during cancer treatment, using antibodies that prevent activation of IRs to restore T_EFF_ functions in TILs (130). Tumors are usually populated with heterogeneous populations of TILs that exhibit varying degrees of dysfunctionality (131). A study that divided TILs into subsets based on their response to antigen stimulation found that the optimal targets of ICB therapy were partially differentiated progenitor cells, that had limited proliferative potential or functional capacity, and CTLs that were programmed for a robust lytic response. Conversely, terminally differentiated CTLs that did not produce cytokines or undergo homeostatic proliferation were poor targets for ICB therapy (132). Development of ICB therapy led to major advances in cancer treatment and improved outcomes for patients with several different types of cancer including metastatic melanoma. The suppressive properties of TGFβ can undermine the effects of ICB therapy by enhancing functional defects in TILs and upregulating hypoxia genes (133, 134). Some cancer treatments use combined strategies to augment CTL responses such as inhibition of PGSL-1 signaling during PD-1 blockade (32). Other cancer treatments utilize agonist antibodies that recognize costimulatory molecules to promote T cell proliferation, survival and memory formation (135). One study found that combined treatment with anti-OX40/anti-CTLA4 reduced IRs expression on tumor specific CTLs and enhanced cytokine production (135), while others found that simultaneous blockade of TGFβ and PD-L1 increased CTL infiltration in tumors of patients with metastatic urothelial cancer (136).

TGFβ alters T cell migration using a variety of direct and indirect mechanisms. A study found that treatment with CXCL12 prompted CTLs to leave cutaneous tumors via lymphatic vessels (137), whereas suppression of CXCL12 expression in senescent tumor cells enhanced T cell infiltration in colonic tumors (138). A third study found that the metastatic properties of mesenchymal cancer cells decreased after CXCL12 was downregulated by TGFβ (139). Others analyzed tumors in breast cancer patients and found that clusters of quiescent cancer cells created an immunosuppressive environment and prevented immune cell infiltration into the tumor by initiating a hypoxia program (140). Another breast cancer study found that hypoxic conditions induced multiple EMT-related pathways including TGFβ (141). CD39 is an ectoenzyme that contributes to immune dysfunction by increasing the concentrations of extracellular adenosine in the tumor microenvironment. Dual CD39 and CD103 expression is a distinguishing feature of TILs in patients with colorectal cancer (142). CD39 and CD73 blockade has been used to enhance CTL responses in tumors. A study found that a TGF-β/SOX4 signaling pathway acted in combination with ROS-driven autophagy to induce CD39 expression on Tregs (143).

## TGFβ plays a role in regulation of cell-fate determining transcription factors

Tbet and eomesodermin (EOMES) are homologous Tbox transcription factors that have cooperative functions during lineage-specification of newly activated and exhausted CTLs (58, 144). T-bet is induced by IL-12 soon after antigen stimulation and plays a key role T_EFF_ formation (145), whereas EOMES is expressed with slightly delayed kinetics and cooperates with T-bet to facilitate formation of T_CM_ cells. Both transcription factors are downregulated as T_RM_ cells settle in peripheral tissues (146). Homologous zinc-finger E homeobox-binding proteins (ZEB1 and ZEB2) are also transcription factors that have important functions during EMT and immune cell development (147). During CD8 T cell differentiation, ZEB2 cooperates with T-bet to promote formation of terminally differentiated T_EFF_ cells, while repressing genes that support T_CM_ development (74). KLRG1^+^ CTLs express ZEB2 at high levels, whereas ZEB1 is induced by TGFβ and promotes formation of T_CM_ cells by counteracting the effects of ZEB2 (148).

During memory formation, TGFβ blocks IL-12 signaling by inhibiting tyrosine phosphorylation and activation of Jak-2 and Tyk-2 kinases (65). EOMES is down regulated by IL-12 during T_EFF_ formation and TGFβ during T_RM_ development (9, 149), whereas expression was maintained in exhausted CTLs (150). EOMES binding sites in the regulatory regions for several genes that encode IRs including PD1, CTLA-4 and CD39 (151). EOMES expression can be upregulated by several different mechanisms during the CTL response, including the antigen receptor (TCR), NF-κB, and selected cytokines. Common gamma chain cytokines are T cell differentiation factors that signal via STAT5 (152). The genes that encode EOMES and TGFβR2 contain STAT5 binding sites. CD8 T cells that were modified to express a constitutively actively form of STAT5 expressed EOMES at increased levels, whereas TGFβR2 was downregulated (153).

Chronic antigen stimulation is largely responsible immune dysfunction during persistent infections and tumorigenesis. Exhausted CTLs can be compartmentalized into subsets based on distinct transcriptional profiles. Definitive markers include T cell factor 1 (TCF-1), which is expressed at high levels in self-renewing CTLs and down regulated during formation of terminally exhausted CTLs that express PD-1 and Tim3 (154). Some TCF-1^+^ CTLs become functional T_EFF_ cells during ICB (155). Lineage tracing was used to track clonal populations of CTLs during LCMV infection and identified a subset of partially differentiated CTLs that segregated between two bifurcating differentiation pathways to create distinct subsets of terminally differentiated and exhausted CTLs (156). Exhaustion was linked to an IRF7-dependent mechanism that was activated by type I IFN, whereas ZEB2 promoted T_EFF_ formation.

Epigenetic programming limits T cell proliferation during ICB therapy. Thymocyte selection-associated high mobility group box (TOX) is a transcription factor that plays a role in formation of exhausted CTLs and positively correlates with IR expression. A recent study showed that stimulation with TGFβ prolonged survival of chronically activated CTLs by attenuating TCR signaling, while inducing epigenetic changes that accelerated terminal dysfunction and attenuated TOX expression (157). Blocking TGFβ signaling in the presence of BMP4 altered the epigenetic state of dysfunctional T cells and restored some T_EFF_ functions. Similarly, rebalancing TGFβ1/BMP signaling during LCMV infection was sufficient to boost CTL responses during PD-L1 blockade and decrease in viral titers (157).

## A TGFβ independent role for SMAD4 during formation of terminally differentiated CTLs

During recent years, multiple groups used Loxp recombination to prevent TGFβ receptor (TGFβRII) and/or SMAD4 expression in CTLs. Phenotypic changes that were observed after antigen stimulation showed that multiple homing receptors were cooperatively regulated by alternative signaling pathways. Notably, signaling via SMAD4 altered the expression levels several homing receptors via a mechanism that did not involve TGFβ (9, 158, 159). By comparing the transcriptomes of T_EFF_ cells, we showed that TGFβ and SMAD4 coordinate changes in homing receptor expression by altering the expression levels of the same genes in opposite directions (9, 10). The target genes included a collection of adhesion molecules (KLRG1, CD62L, CD103) and transcription factors (Hobit and EOMES) with important functions during memory formation (9). The SMAD4-deficient CTLs expressed EOMES at reduced levels (9), similar to the pattern seen in stem-like memory CD8 T cells during chronic antigen stimulation (9). The regulatory sequences in the EOMES promoter contain multiple SMAD4 binding sites (9, 160). The SMAD4-deficient CTLs expressed CX_3_CR1 at low/intermediate levels (9), while KLRG1 and CD62L were down regulated indicating a defect during formation of terminally differentiated T_EFF_ cells and T_CM_ cells (10, 11). Ectopic EOMES expression induced phenotypic changes that were consistent with a shift toward T_CM_ formation (9).

## Concluding remarks

Although TGFβ is a key regulator during the CTL response (**Table 1**), the pleiotropic effects of the cytokine cause complications during immune intervention. The recent discovery that SMAD4 plays a separate role in lineage-specification of newly activated CTLs, via a mechanism that does not involve TGFβ, may reveal new avenues for augmenting CTL responses after vaccination. For example, it may be possible to generate inhibitors that prevent formation of terminally differentiated CTLs and enhance formation of CTLs with stem-like properties that localize to tumors. Since TCR stimulation in the presence of recombinant IL-2 and IL-12 is not sufficient to induce KLRG1 expression on CTLs *in vitro *(81, 111, 161), current data indicate that multiple stimuli are required for terminal differentiation including inflammatory molecules that signal via SMAD4. Additional work is required to identify of the ligand(s) of this novel regulatory pathway and determine whether signaling induces epigenetic that make terminally differentiated T_EFF_ and T_CM_ refractory to subsequent regulation by TGFβ. Several groups examined the functions of SMAD4 during CD8 T cell differentiation. Variations in the results between studies may reflect the timing of Cre expression. Some studies found that SMAD4-ablation during an early stage of thymic development altered homing receptor expression, as well as cell-proliferation and effector functions (10, 159). Others found that SMAD4-deficient CTLs maintained normal effector functions and proliferation when the mutation occurred immediately before CTLs left the thymus (9, 11). Since KLRG1 is an inhibitory receptor that binds N- cadherin, further work is required to reveal whether interactions between N-cadherin and KLRG1 prevent T_EFF_ cells from extravasating to the periphery during tissue repair.

**Table 1:** Table of regulatory pathways.

		Naïve	Activated	SLECs	T_CM_	T_RM_
**Cellular Processes**	**TGFβ**	Activation (ref 24)	Proliferation (ref 11)	Proliferation (ref 11)	Cell Survival (ref 87)	Development, Maintenance (ref 149)
				Apoptotic cell death (ref 82)		
	**Smad4**		Differentiation (ref 10,11)	Differentiation (ref 10, 11)		Differentiation (ref 9, 11)
**Regulated genes**	**TGFβ**	Itgae (CD103) (ref 14)		Bcl2 (ref 82)	Bcl2 (ref 87)	Itgae (CD103) (ref 23, 149)
			Zeb1 (ref 148)		Zeb1 (ref 148)	
			Zeb2 (ref 148)		Zeb2 (ref 148)	
			KLRG1 (ref 120)	Granzyme B (ref 87)	T-bet (ref 87)	T-bet (ref 146)
			VISTA (ref 30)	CXCR3 (ref 101)	Eomes (ref 87)	Eomes (ref 146)
			PSGL-1 (33)*		Foxo1, Bcl6 (ref 87)	S1PR5, KLF2 (refs 113, 114)
	**Smad4**	Itgae (CD103) (ref 9)		KLRG1 (ref 11)	Sell (CD62L) (ref 11)	Itgae (CD103) (ref 9)
				CX3CR1 (ref 9)	Eomes (ref 9)	Hobit (ref 9)

*Only confirmed in CD4 T cells.

White boxes indicate positive regulation by TGFβ. Grey boxes indicate negative regulation by TGFβ.

## Author contributions

This review was written by LC and KC. All authors contributed to the article and approved the submitted version.
